# Surgical Cases in Private Practice of Dr. C. C. F. Gay

**Published:** 1881-07

**Authors:** 


### Clinical Reports.

### SURGICAL CASES IN PRIVATE PRACTICE OF DR. C. C. F. GAY.

### DORSAL DISLOCATION OF THE HEAD OF THE LEFT FEMUR OF FOUR WEEKS' DURATION—REDUCTION BY THE FLEXION METHOD.

Charles Palmer, aged 26 years, very muscular, weight 240 pounds, a brakeman on the Erie Railway, was descending a ladder on January 29, 1881, at the rear end of a car, when the engine was reversed, which caused the man to be caught between two cars. He was struck in his back when his left thigh was flexed at a right angle with his body; his foot being upon the round of the ladder. Dr. Armstrong was at once called to attend the patient, etherized him, manipulated his limb and left him, believing he had succeeded in reducing a luxation of the hip. Undoubtedly reduction was effected; no retaining apparatus was applied. At length the Doctor, observing much shortening of the limb, called Dr. Dorr in consultation. At their visit, the diagnosis being involved in doubt, my presence was requested. Accordingly, on February 25, we visited the patient together. At this visit I learned that the patient had been walking about, at times, considerable distances from home with the aid of crutches; that he had suffered but little pain after the first few days; that now, when standing or lying down the limb was inverted, adducted, semi-flexed; two inches shortened, and the patient was content to have it let alone; Nelaton's line fell two inches below the great trochanter; diagnosis of dorsal dislocation was readily made out.

The patient was placed upon a settee, chloroformed by Dr. Armstrong, and Dr. Dorr and myself proceeded to the reduction by the flexion method. After spending a little time and force in breaking up supposed adhesions, the limb was flexed upon the abdomen, abducted, rotated inward, and extended. After two or three trials in this way, Dr. Dorr, in the course of twelve or fifteen minutes, succeeded in reducing the luxation. To make it sure that reduction had been accomplished, I next took up the limb, flexed the thigh to about a right angle with the body, when the head of the femur was plainly felt to leave its socket again. I now repeated the former maneuver and readily reduced the dislocation. Its reduction was manifest by the peculiar sound which was quite audible and also by the correction of deformity. Upon measurement of the limbs they were found to be of equal length, viz: 34½ inches; inversion, adduction and flexion were overcome and Nelaton's line fell above the trochanter. The limb was secured by adhesive straps and bandage around the pelvis and over the trochanters, the knees and feet were fastened together and extension employed by weight and pulleys.

March 10th all appliances were removed and patient allowed to get around on crutches.

May 21. Recovery perfect; patient knows no difference between the two limbs; strength and usefulness of both are alike, and there is no deformity.

---

POPLITEAL ANEURISM, TREATMENT FOR EIGHT DAYS BY COMPRESSION WITH THE ELASTIC BANDAGE; LIGATION OF THE SUPERFICIAL FEMORAL ARTERY; SECONDARY HEMORRHAGE; RECOVERY.

JNO. COGAN, age 30 years; by occupation a coal shoveler; previous health good. Six weeks since he began to go lame and supposed he had rheumatism at the knee-joint; could scarcely bend the knee; it was swollen and painful. Dr. Vaughn was consulted on Feb. 28, 1881. He discovered a tumor at the flexure of the knee which he diagnosed aneurism. The tumor was about the size of a large goose egg and quite prominent at the flexure and outer side of the knee. It is supposed to have been caused by pressing the knee against the hand or handle of the shovel in shoveling coal. The patient was put to bed, and Tr. Iodine with the rubber bandage over a compress were employed for eight days; the bandage was applied from the foot up above the tumor and resulted, the doctor thinks, in diminishing, somewhat, the size of the tumor. On March 2d Dr. Vaughn called myself in consultation. Upon examination I find that the tumor is large, elastic, compressible and pulsates. Pressure upon the femoral artery stops the pulsation. Bruit is not heard except when the leg is flexed nearly to a right angle, when it becomes very distinct. What is very unusual, the patient suffers now no pain. The limb is semi-flexed. To-day, March 3d, the patient was etherized. Assisted by Drs. Vaughn, Bartow, Pettit, and Guess, I ligated the superficial femoral artery four and a half inches below Poupart's ligament by measurement, or two and a half inches below the deep femoral, using a large carbolized silk ligature. Pulsation, in the tumor, ceased at once upon tightening the ligature. Measurement, immediately after the operation, around the limb over the patella showed sixteen and a half inches, and the sound limb fourteen inches.

The wound was closed by four silk sutures, the limb enveloped in cotton batting, several bottles of hot water applied and patient directed to keep his bed.

March 4th, pulse 100; temperature 100 in axilla; temperature 100 in popliteal space; temperature 99 in popliteal sound side.

No pain, numbness, or sensation of pricking.

March 5th, pulse 98; temperature at axilla 99¾; temperature popliteal space both limbs 98½.

March 6th, temperature at every point 98½.

March 7th, temperature at axilla 98½; temperature at popliteal spaces 98½; pulse 88; no complaint of patient from any cause.

March 11th, secondary hemorrhage occurred which was quite profuse for a moment, but it had been anticipated and provisions made to arrest it by loosely adjusting, two days previously, a tourniquet to the limb, which was tightened by the patient himself.

The wound was opened and the ligature found in situ around the artery. Pressure was now removed and hemorrhage shown to have ceased. Accordingly I plugged the wound with small pieces of sponges dipped into sol. persulph ferri, diluted one-half with water, and applied compress and bandage.

March 14th. Dressing and sponges were removed and the wound irrigated with carbolized water. There is no hemorrhage. Again dressed with compress and bandage, continuing in use the tourniquet. There is a little œdema about the ankles. Pulse 100.

March 17th. While dressing the wound Dr. Vaughn brought away the ligature.

March 22d. Circumference of limb one inch less.

June 18th. Patient returned to work, having no indications of recurrence of the tumor. Both limbs at knee measure alike, viz., 14½ inches.

